# Developing and Validating for Cognitive Screening Tools for Identifying and Intervening Dementia among Older Persons in Rural Uganda.

**DOI:** 10.1192/j.eurpsy.2023.417

**Published:** 2023-07-19

**Authors:** D. Jephthah, I. Ddumba

**Affiliations:** Nursing, Victoria University, Kampala, Uganda

## Abstract

**Introduction: Background:**

Although risk of developing dementia increase in later years, identification and assessment of older persons with dementia in developing countries is still low. Access to easy and user friendly cognitive screening tools by the health care professional in developing countries is difficult.

**Objectives:**

The study aimed to develop, validate and field test the cognitive screening tool for use in outpatient departments within health facilities in Uganda.

**Methods:**

In the rural eastern region of Uganda, twenty-three (23) purposively selected health facilities and administered a scientifically derived cognitive screening tools to all eligible older persons. We conducted an inter-rater reliability in all the health facilities using three raters. Diagnosis of dementia (DSM-IV) was classified as a major cognitive impairment and was quality checked by physiatrist who were blinded to results of the screening assessment.

**Results:**

The area under the receiver operating characterizes (AUROC) curve in health facilities was 0.912. The inter-rater reliability was good (Intra-class correlation coefficient of 0.692 to 0.734). the predictive accuracy of the tool to discriminate between dementia and other cognitive impairment was 0.892. In regression modal, the cognitive screening tool, didn’t appear to be biased by age.

**Conclusions:**

The cognitive screening tool if performed well among the older persons, can be proved useful for screening dementia in other developing countries

**Disclosure of Interest:**

None Declared

